# Supplementary material for: Suicide fatalities in the US compared to Canada: Potential suicides averted with lower firearm ownership in the US
Source: PLoS One. 2020 Apr 30;15(4):e0232252. doi: 10.1371/journal.pone.0232252 (PMC7192495; doi:10.1371/journal.pone.0232252)
Supplement: S1 Text — (DOCX) [file pone.0232252.s001.docx]

**Text S1. Detailed summary of methods:**

To compare US and Canadian suicide rates while accounting for ethnicity, we created a “Standardized US population” matching the ethnic composition of the Canadian population. We describe the steps we took to standardize the data and produce estimated suicide fatalities averted below.

1. We downloaded demographic data on ethnicity by sex-specific age groups in Canada from two sets of tables from the 2016 Canadian Census, through Statistics Canada: (1) a set of detailed ethnicity tables listing the number of people who reported each continent and country of origin for each sex-specific age group and whether individuals reported the relevant country of origin as a single or multiple country of origin response,^2^ and (2) a less-detailed set of tables listing the number of people who reported Aboriginal (First Nations, Métis, and Inuit) and non-Aboriginal origin, for each sex-specific age group. This table also listed the number of people for whom each Aboriginal origin response was a single response or a multiple origin response.^3^ We used these two tables to develop the ethnicity weights to apply to the US population and suicide fatality rates to make them more comparable to Canada.

2. We used the “Total - residence on or off reserve” column of the less detailed ethnicity table to extract the number of people with any Aboriginal ethnicity in each sex-specific age group. We added the “Aboriginal ancestry (only)” cell and the “Aboriginal and non-Aboriginal ancestries” cell, categorizing any people with any aboriginal ancestry as aboriginal.

3. We estimated the number of Canadians likely to be Black in each sex-specific age group using the “African origins” cell of the “Total - single and multiple ethnic origin responses” column from the more detailed table of ethnicities, meaning that we considered all those who reported any African origins Black.

4. For each sex-specific age group, we estimated the number of non-Aboriginal, non-African Canadians by subtracting the Aboriginal and African numbers from the total denominator of those who reported ethnicities, as reported in the less detailed ethnicity table, as can be seen in the third row of Table 1.
