# Supplementary material for: Suicide fatalities in the US compared to Canada: Potential suicides averted with lower firearm ownership in the US
Source: PLoS One. 2020 Apr 30;15(4):e0232252. doi: 10.1371/journal.pone.0232252 (PMC7192495; doi:10.1371/journal.pone.0232252)
Supplement: S1 Table — (DOCX) [file pone.0232252.s002.docx]

Table S1. Canadian ethnicity distribution as estimated through the 2016 Canadian Census, among males aged 0-14.

|  |  |
| --- | --- |
| **Ethnicity** | **Population** |
| Aboriginal | 101950 + 185520 = 287470 |
| African origins | 161155 |
| Non-aboriginal, non-African | 2981145 - 287470 - 161155 = 2532520 |
| **Total** | 2981145 |

Note: The total numbers of individuals represented in the two different sets of demographic tables stratified by age and sex sometimes differed by 5-10 individuals, due to random rounding (by multiples of 5) to preserve confidentiality in smaller groups, as described on the Statistics Canada website and through correspondence with the group. There were also differences in the number of Canadians who reported their ethnicity relative to the numbers listed in suicide fatality data because the ethnicity questions were part of the long-form census which was only administered to private households, and thus does not include collective dwellings. These differences were present overall, with 34,460,065 observations of ethnicity and 35,151,730 people in the population, and in sex-specific age groups.

5. We downloaded data from the Web-based Injury Statistics Query and Reporting System (WISQARS) through the Centers for Disease Control and Prevention (CDC)^1^ on firearm and non-firearm suicide deaths within age, sex, and ethnic groups in the US, to standardized these data.

6. To standardize suicide rates using Canadian ethnic distributions within each sex-specific age group, we approximated Canadian ethnicity groups using US ethnic groups in the WISQARS death data, within the same sex-specific age groups for which Statistics Canada provided data. We approximated the “African” category using the “Black” category in the US data and the “Aboriginal” category using the “American Indian/Alaska Native” category in the US data, as seen in Table S2 below for US deaths in males aged 0-14. For each sex-specific age group, we combined the death counts and population denominators in the “Asian/Pacific Islander” group with those in the “White” group, to approximate a “non-Aboriginal, non-African” group, as seen in the third row of the table. We obtained total suicide-fatality and cause-specific suicide fatality data in the sex-specific age groups by these three ethnicity categories.
