# Supplementary material for: Suicide fatalities in the US compared to Canada: Potential suicides averted with lower firearm ownership in the US
Source: PLoS One. 2020 Apr 30;15(4):e0232252. doi: 10.1371/journal.pone.0232252 (PMC7192495; doi:10.1371/journal.pone.0232252)
Supplement: S2 Table — (DOCX) [file pone.0232252.s003.docx]

Table S2. Firearm and non-firearm suicide deaths and denominators for the US in 2016 according to WISQARS among males aged 0-14, grouped into ethnicity categories to align with Canada.

| **Ethnicity** | **Number of firearm suicides** | **Number of non-firearm suicides** | **Population** |
| --- | --- | --- | --- |
| Any Aboriginal origins | 3 | 4 | 585881 |
| Any African origins | 10 | 22 | 5196385 |
| Non-aboriginal, non-African | 110 + 3 = 113 | 112 + 8 = 120 | 23396259 + 1956983 = 25353242 |
| **Total** | 126 | 146 | 31135508 |

7. Within each sex-specific age group, we then multiplied the proportion of the Canadian population reporting each ethnic group (as in Table S1) by the crude suicide rate for the corresponding ethnic group in the US (as in Table S2), and summed across ethnic groups.

For example, the equations for standardized firearm suicide deaths in the US were as follows:

(proportion Aboriginal in Canada  x Aboriginal firearm suicide rate in US) +

(proportion African in Canada x African firearm suicide rate in US)  +

(proportion other in Canada x Other firearm suicide rate in US))

8. We multiplied each calculated, sex-specific, age-specific rate by 100,000 to get the standardized number of deaths per 100,000 population in each group.

9. Within each sex-specific age group, we summed together standardized firearm and non-firearm rates (as calculated above) to get the total standardized suicide rates per 100,000.

Specifically, for males aged 0 to 14, steps 7-9 are shown in Table S3.
