# Supplementary material for: Suicide fatalities in the US compared to Canada: Potential suicides averted with lower firearm ownership in the US
Source: PLoS One. 2020 Apr 30;15(4):e0232252. doi: 10.1371/journal.pone.0232252 (PMC7192495; doi:10.1371/journal.pone.0232252)
Supplement: S3 Table — (DOCX) [file pone.0232252.s004.docx]

Table S3. Calculated firearm and non-firearm suicide rates in the US, standardized to the ethnic distribution of Canada, 2016, males aged 0-14.

| **Firearm suicide rate per 100,000** | **Non-firearm suicide rate per 100,000** | **Total suicide rate per 100,000** |
| --- | --- | --- |
| (((287470 / 2981145) x (3 / 585881)) +  ((161155 / 2981145) x (10 / 5196385)) +  ((2532520 / 2981145) x (113 / 25353242)))  x 100,000  = 0.4384 | (((287470 / 2981145) x (4 / 585881)) +  ((161155 / 2981145) x (22 / 5196385)) +  ((2532520 / 2981145) x (120 / 25353242)))  x 100,000  = 0.4908 | 0.4384 +  0.4908 =  0.9292 |

10. We calculated the potential rates of suicide fatalities averted if the US had the same suicide rates as in Canada by subtracting the Canadian suicide fatality rates from the US standardized suicide fatality rates in each sex-specific age group, for overall suicide fatalities and for firearm-specific suicide fatalities. For non-firearm suicides, since the Canadian rates were higher than US rates, we subtracted the standardized US suicide fatality rates from the Canadian suicide fatality rates, in order to calculate the rates of potential *additional* non-firearm deaths, instead of the rates of deaths averted.

11. We estimated the age- and sex-specific proportions of suicide fatalities that would be averted (or proportional increases, for non-firearm deaths) by dividing the rate differences (from step 10) in each sex-specific age group by the US standardized rates of suicide fatalities in each sex-specific age group, overall and by cause.

12. We estimated the numbers of US suicide fatalities that would be averted (or number of additional deaths, for non-firearm deaths) by multiplying the proportion of suicide fatalities averted in each sex-specific age group (from step 11) by the number of observed (actual) US suicide fatalities in each sex-specific age group, and then summing across sex-specific age groups. We did this for overall suicide fatalities and for cause-specific suicide fatalities separately.

As an example, we depict steps 10-12 in Table S4 for all-cause suicides in males aged 0 to 14.
