# Supplementary material for: Suicide fatalities in the US compared to Canada: Potential suicides averted with lower firearm ownership in the US
Source: PLoS One. 2020 Apr 30;15(4):e0232252. doi: 10.1371/journal.pone.0232252 (PMC7192495; doi:10.1371/journal.pone.0232252)
Supplement: S4 Table — (DOCX) [file pone.0232252.s005.docx]

Table S4. All-cause suicide deaths in the US that would be averted if the US had the same suicide rates as in Canada, 2016, among males aged 0-14.

| **Standardized US suicide rate per 100,000** | **Canadian suicide rate per 100,000** | **Difference in rates (rate averted per 100,000)** | **Proportional difference (difference in rates divided by the US standardized rate)** | **Number of observed US deaths** | **Estimated number of deaths averted** |
| --- | --- | --- | --- | --- | --- |
| 0.9292 | 0.5346 | 0.9292 - 0.5346 = 0.3946 | 0.3946 / 0.9292 = 0.4247 | 272 | 272 x 0.4247 = 115.5 |

13. In order to calculate the total proportion of deaths averted (for all suicide deaths and firearm-specific suicide deaths) or total proportional increases (for non-firearm suicide deaths), we divided the number of deaths averted (or additional deaths) by the actual observed number of suicides in the US in each category, for men, women, and overall.

For example, for firearm suicide deaths among men, the sum across age groups for potential number of deaths averted was 9992.17, which we then divided by the observed number of firearm suicide deaths in the US among men of all age groups (34,725) to get 0.288, or roughly a 29% decrease in firearm deaths among men if the US had the same rate of firearm deaths as in Canada.
