# Supplementary material for: Suicide fatalities in the US compared to Canada: Potential suicides averted with lower firearm ownership in the US
Source: PLoS One. 2020 Apr 30;15(4):e0232252. doi: 10.1371/journal.pone.0232252 (PMC7192495; doi:10.1371/journal.pone.0232252)
Supplement: S5 Table — (DOCX) [file pone.0232252.s006.docx]

Table S5. Firearm and non-firearm suicide deaths, population, and crude rates in Canada, according to Statistics Canada,^4,5^ by age and sex, 2016.

| **Age group** | **Sex** |  | **Firearm suicides** | **Firearm suicide rate per 100,000** | **Non-firearm suicides** | **Non-firearm suicide rate per 100,000** | **Total suicides** | **Total suicide rate per 100,000** |
| --- | --- | --- | --- | --- | --- | --- | --- | --- |
| **0 to 14** | **Male** | No. deaths | 1 | 0.033412 | 15 | 0.50118279 | 16 | 0.534595 |
|  |  | Population | 2992920 |  | 2992920 |  | 2992920 |  |
|  | **Female** | No. deaths | 0 | 0 | 32 | 1.12413034 | 32 | 1.12413 |
|  |  | Population | 2846645 |  | 2846645 |  | 2846645 |  |
| **15 to 24** | **Male** | No. deaths | 52 | 2.381269 | 293 | 13.4175325 | 345 | 15.7988 |
|  |  | Population | 2183710 |  | 2183710 |  | 2183710 |  |
|  | **Female** | No. deaths | 1 | 0.047958 | 141 | 6.76211966 | 142 | 6.810078 |
|  |  | Population | 2085145 |  | 2085145 |  | 2085145 |  |
| **25 to 34** | **Male** | No. deaths | 59 | 2.573318 | 383 | 16.7047576 | 442 | 19.27808 |
|  |  | Population | 2292760 |  | 2292760 |  | 2292760 |  |
|  | **Female** | No. deaths | 7 | 0.301382 | 153 | 6.58736002 | 160 | 6.888743 |
|  |  | Population | 2322630 |  | 2322630 |  | 2322630 |  |
| **35 to 44** | **Male** | No. deaths | 65 | 2.923878 | 433 | 19.4775255 | 498 | 22.4014 |
|  |  | Population | 2223075 |  | 2223075 |  | 2223075 |  |
|  | **Female** | No. deaths | 2 | 0.086191 | 148 | 6.37814194 | 150 | 6.464333 |
|  |  | Population | 2320425 |  | 2320425 |  | 2320425 |  |
| **45 to 54** | **Male** | No. deaths | 103 | 4.159079 | 512 | 20.6742553 | 615 | 24.83333 |
|  |  | Population | 2476510 |  | 2476510 |  | 2476510 |  |
|  | **Female** | No. deaths | 4 | 0.156157 | 221 | 8.62765613 | 225 | 8.783813 |
|  |  | Population | 2561530 |  | 2561530 |  | 2561530 |  |
| **55 to 64** | **Male** | No. deaths | 111 | 4.624865 | 428 | 17.8328132 | 539 | 22.45768 |
|  |  | Population | 2400070 |  | 2400070 |  | 2400070 |  |
|  | **Female** | No. deaths | 7 | 0.278808 | 170 | 6.77106049 | 177 | 7.049869 |
|  |  | Population | 2510685 |  | 2510685 |  | 2510685 |  |
| **65+** | **Male** | No. deaths | 153 | 5.676864 | 327 | 12.1329054 | 480 | 17.80977 |
|  |  | Population | 2695150 |  | 2695150 |  | 2695150 |  |
|  | **Female** | No. deaths | 5 | 0.154298 | 148 | 4.56721756 | 153 | 4.721515 |
|  |  | Population | 3240485 |  | 3240485 |  | 3240485 |  |
