# Supplementary material for: Suicide fatalities in the US compared to Canada: Potential suicides averted with lower firearm ownership in the US
Source: PLoS One. 2020 Apr 30;15(4):e0232252. doi: 10.1371/journal.pone.0232252 (PMC7192495; doi:10.1371/journal.pone.0232252)
Supplement: S6 Table — (DOCX) [file pone.0232252.s007.docx]

Table S6. Firearm and non-firearm suicide deaths, population, and crude rates in the US, standardized to the ethnic distribution of Canada, by age and sex, 2016.

| **Age group** | **Sex** |  | **Firearm suicides** | **Firearm suicide rate per 100,000** | **Non-firearm suicides** | **Non-firearm suicide rate per 100,000** | **Total suicides** | **Total suicide rate per 100,000** |
| --- | --- | --- | --- | --- | --- | --- | --- | --- |
| **0 to 14** | **Male** | No. deaths | 13.06961 | 0.4384092 | 14.631662 | 0.4908068 | 27.70128 | 0.929216 |
|  |  | Population | 2981145 |  | 2981145 |  | 2981145 |  |
|  | **Female** | No. deaths | 3.372546 | 0.1189233 | 14.807915 | 0.5221593 | 18.18046 | 0.641083 |
|  |  | Population | 2835900 |  | 2835900 |  | 2835900 |  |
| **15 to 24** | **Male** | No. deaths | 237.1276 | 10.925526 | 241.69841 | 11.1361228 | 478.8260 | 22.06165 |
|  |  | Population | 2170400 |  | 2170400 |  | 2170400 |  |
|  | **Female** | No. deaths | 33.0271 | 1.6022256 | 91.010604 | 4.41515063 | 124.0377 | 6.017376 |
|  |  | Population | 2061325 |  | 2061325 |  | 2061325 |  |
| **25 to 34** | **Male** | No. deaths | 289.6842 | 12.789787 | 333.43800 | 14.7215522 | 623.1222 | 27.51134 |
|  |  | Population | 2264965 |  | 2264965 |  | 2264965 |  |
|  | **Female** | No. deaths | 53.0271 | 2.2939448 | 116.73585 | 5.04998024 | 169.7629 | 7.343925 |
|  |  | Population | 2311610 |  | 2311610 |  | 2311610 |  |
| **35 to 44** | **Male** | No. deaths | 288.4995 | 13.143065 | 318.57623 | 14.5132606 | 607.0757 | 27.65633 |
|  |  | Population | 2195070 |  | 2195070 |  | 2195070 |  |
|  | **Female** | No. deaths | 66.98394 | 2.8963524 | 146.49207 | 6.33424429 | 213.4760 | 9.230597 |
|  |  | Population | 2312700 |  | 2312700 |  | 2312700 |  |
| **45 to 54** | **Male** | No. deaths | 384.8301 | 15.754549 | 372.78564 | 15.2614626 | 757.6157 | 31.01601 |
|  |  | Population | 2442660 |  | 2442660 |  | 2442660 |  |
|  | **Female** | No. deaths | 89.18712 | 3.4984746 | 193.13554 | 7.57597785 | 282.3227 | 11.07445 |
|  |  | Population | 2549315 |  | 2549315 |  | 2549315 |  |
| **55 to 64** | **Male** | No. deaths | 430.7349 | 18.206272 | 293.3547 | 12.3994952 | 724.0896 | 30.60577 |
|  |  | Population | 2365860 |  | 2365860 |  | 2365860 |  |
|  | **Female** | No. deaths | 81.99228 | 3.2939210 | 169.41821 | 6.80613089 | 251.4105 | 10.10005 |
|  |  | Population | 2489200 |  | 2489200 |  | 2489200 |  |
| **65+** | **Male** | No. deaths | 642.8741 | 25.196124 | 184.65460 | 7.23715663 | 827.5287 | 32.43328 |
|  |  | Population | 2551480 |  | 2551480 |  | 2551480 |  |
|  | **Female** | No. deaths | 59.39712 | 2.0282922 | 105.57973 | 3.60533555 | 164.9768 | 5.633628 |
|  |  | Population | 2928430 |  | 2928430 |  | 2928430 |  |
