# Supplementary material for: Suicide fatalities in the US compared to Canada: Potential suicides averted with lower firearm ownership in the US
Source: PLoS One. 2020 Apr 30;15(4):e0232252. doi: 10.1371/journal.pone.0232252 (PMC7192495; doi:10.1371/journal.pone.0232252)
Supplement: S7 Table — (DOCX) [file pone.0232252.s008.docx]

Table S7. Firearm and non-firearm suicide deaths, denominators, and crude rates for the US according to WISQARS,^1^ by age and sex, 2016, grouped into ethnicity categories to align those provided for the Canadian population.

| **FIREARM SUICIDE** | | | | | | | |
| --- | --- | --- | --- | --- | --- | --- | --- |
|  |  |  | **Ethnicity** | | |  |  |
| **Age Group** | **Sex** |  | **Any Aboriginal origins** | **Any African origins** | **Non-aboriginal, non-African** | **Total firearm suicides** | **Total firearm suicide rates per 100,000** |
| **0 to 14** | **Male** | No. deaths | 3 | 10 | 113 | 126 | 0.4046827 |
|  |  | Population | 585881 | 5196385 | 25353242 | 31135508 |  |
|  | **Female** | No. deaths | 0 | 0 | 34 | 34 | 0.1139427 |
|  |  | Population | 570162 | 5027401 | 24241998 | 29839561 |  |
| **15 to 24** | **Male** | No. deaths | 46 | 305 | 1997 | 2348 | 10.532496 |
|  |  | Population | 389861 | 3688506 | 18214544 | 22292911 |  |
|  | **Female** | No. deaths | 4 | 42 | 289 | 335 | 1.5788395 |
|  |  | Population | 374352 | 3560736 | 17283028 | 21218116 |  |
| **25 to 34** | **Male** | No. deaths | 50 | 341 | 2427 | 2818 | 12.469171 |
|  |  | Population | 374596 | 3338882 | 18886260 | 22599738 |  |
|  | **Female** | No. deaths | 10 | 54 | 416 | 480 | 2.1741587 |
|  |  | Population | 346167 | 3466922 | 18264416 | 22077505 |  |
| **35 to 44** | **Male** | No. deaths | 29 | 171 | 2344 | 2544 | 12.623579 |
|  |  | Population | 309569 | 2702577 | 17140617 | 20152763 |  |
|  | **Female** | No. deaths | 7 | 37 | 511 | 555 | 2.7316497 |
|  |  | Population | 297098 | 3017848 | 17002447 | 20317393 |  |
| **45 to 54** | **Male** | No. deaths | 21 | 135 | 3005 | 3161 | 14.976685 |
|  |  | Population | 277098 | 2666665 | 18162376 | 21106139 |  |
|  | **Female** | No. deaths | 2 | 22 | 688 | 712 | 3.284051 |
|  |  | Population | 277646 | 3018481 | 18384413 | 21680540 |  |
| **55 to 64** | **Male** | No. deaths | 27 | 122 | 3269 | 3418 | 17.090822 |
|  |  | Population | 216738 | 2284809 | 17497491 | 19999038 |  |
|  | **Female** | No. deaths | 4 | 14 | 631 | 649 | 3.0236526 |
|  |  | Population | 235033 | 2712845 | 18516228 | 21464106 |  |
| **65+** | **Male** | No. deaths | 18 | 114 | 5098 | 5230 | 23.998723 |
|  |  | Population | 169296 | 1866064 | 19757466 | 21792826 |  |
|  | **Female** | No. deaths | 2 | 17 | 507 | 526 | 1.9161157 |

Table S7 *continued*. Firearm and non-firearm suicide deaths, denominators, and crude rates for the US according to WISQARS,^1^ by age and sex, 2016, grouped into ethnicity categories to align with those provided for the Canadian population.

| **NON-FIREARM SUICIDE** | | | | | | | |
| --- | --- | --- | --- | --- | --- | --- | --- |
|  |  |  | **Ethnicity** | | |  |  |
| **Age Group** | **Sex** |  | **Any Aboriginal origins** | **Any African origins** | **Non-aboriginal, non-African** | **Total non-firearm suicides** | **Total non-firearm suicides rates per 100,000** |
| **0 to 14** | **Male** | No. deaths | 4 | 22 | 120 | 146 | 0.468918 |
|  |  | Population | 585881 | 5196385 | 25353242 | 31135508 |  |
|  | **Female** | No. deaths | 6 | 16 | 115 | 137 | 0.45912204 |
|  |  | Population | 570162 | 5027401 | 24241998 | 29839561 |  |
| **15 to 24** | **Male** | No. deaths | 81 | 238 | 1908 | 2227 | 9.98972274 |
|  |  | Population | 389861 | 3688506 | 18214544 | 22292911 |  |
|  | **Female** | No. deaths | 40 | 101 | 672 | 813 | 3.83163142 |
|  |  | Population | 374352 | 3560736 | 17283028 | 21218116 |  |
| **25 to 34** | **Male** | No. deaths | 95 | 289 | 2685 | 3069 | 13.5798034 |
|  |  | Population | 374596 | 3338882 | 18886260 | 22599738 |  |
|  | **Female** | No. deaths | 30 | 78 | 891 | 999 | 4.52496783 |
|  |  | Population | 346167 | 3466922 | 18264416 | 22077505 |  |
| **35 to 44** | **Male** | No. deaths | 56 | 190 | 2504 | 2750 | 13.6457716 |
|  |  | Population | 309569 | 2702577 | 17140617 | 20152763 |  |
|  | **Female** | No. deaths | 22 | 65 | 1094 | 1181 | 5.81275363 |
|  |  | Population | 297098 | 3017848 | 17002447 | 20317393 |  |
| **45 to 54** | **Male** | No. deaths | 31 | 136 | 2870 | 3037 | 14.3891784 |
|  |  | Population | 277098 | 2666665 | 18162376 | 21106139 |  |
|  | **Female** | No. deaths | 10 | 51 | 1466 | 1527 | 7.0431825 |
|  |  | Population | 277646 | 3018481 | 18384413 | 21680540 |  |
| **55 to 64** | **Male** | No. deaths | 19 | 84 | 2224 | 2327 | 11.6355597 |
|  |  | Population | 216738 | 2284809 | 17497491 | 19999038 |  |
|  | **Female** | No. deaths | 5 | 45 | 1315 | 1365 | 6.35945424 |
|  |  | Population | 235033 | 2712845 | 18516228 | 21464106 |  |
| **65+** | **Male** | No. deaths | 2 | 49 | 1473 | 1524 | 6.993127 |
|  |  | Population | 169296 | 1866064 | 19757466 | 21792826 |  |
|  | **Female** | No. deaths | 5 | 22 | 897 | 924 | 3.36595235 |
|  |  | Population | 206658 | 2758899 | 24485812 | 27451369 |  |
